# Mechanics and Analysis of Advanced Materials and Structures

**DOI:** 10.3390/ma16052123

**Published:** 2023-03-06

**Authors:** Sanichiro Yoshida, Giovanni Pappalettera

**Affiliations:** 1 Department of Chemistry & Physics, Southeastern Louisiana University, Hammond, LA 70402, USA; 2 Dipartimento di Meccanica, Matematica e Management, Politecnico di Bari, Via Orabona 4, 70125 Bari, Italy

Modern technological development has made the designing and characterization of materials sophisticated. The design concepts used for centuries for macroscopic objects do not necessarily apply to micro or nano-scale counterparts. The elastic modulus of a nano-scale wire can be significantly different from that of the same material at the macroscopic scale. Composite materials comprising multiple materials possessing completely different properties can exhibit unexpected behaviors. 

This situation requires the advancement of diagnostic methods in characterizing material properties. Great efforts are necessary to extend the type of materials and the conditions to make analysis convenient and to improve the figure of merits such as the sensitivity, resolution, non-destructiveness, and rapidness of the measurement. Such advancement can be an extension or a combination of existing techniques or the development of entirely new methodologies. Often, analytical or numerical modeling is necessary to expedite material characterization. Recent software development provides various computational tools, including machine learning and artificial intelligence algorithms, for numerical analysis and modeling.

The present Special Issue is a collection of articles that shed light on the above issues. It has 11 original research papers and one review article. Below we take a quick look at the gist of these articles.

The first four papers in the reference list discuss the mechanical properties of composite materials. 

The vibrational behavior of plates is complex. It has many modes, and the dynamics depend on the boundary conditions and the material’s characteristics. Huang et al. [1] discuss an analytical solution based on the Mindlin plate theory that describes the free vibration of rectangular plates of functionally graded material. They analyzed various combinations of boundary conditions and validated the results via comparison with published results. They tabulated data from this study so that researchers could use it to judge the accuracy of numerical methods.

Fabric reinforcement of materials is another complex process. While the fabric material increases strength, controlling the interface with the matrix material is challenging. Mercedes et al. [2] developed an analytical approach and a numerical simulator to evaluate the strength of concrete beams with various types of fabrics. They successfully parameterized complex strengthening behaviors with a single parameter called the reduction coefficient. They conclude that this strategy simplifies the analytical design of fiber-reinforced cementitious matrices.

In a slightly different context from the above two works, Obrezkov et al. [3] performed numerical modeling. They developed a numerical method to analyze a biomedical composite material. They modeled the elongation-induced deformation of the human Achilles tendon by assuming that it consists of beam elements using absolute nodal coordinate formulation. They report that this method reduces the degree of freedom, benefitting cost reductions in computation. They conclude that with some limitations, this model is feasible.

The aerospace industry requires the development of ultra-high-temperature materials. In ref. [4], Tsakiropoulos analyzed the stability of complex-concentrated and high-entropy Niobium-based solid solutions for ultra-high temperature materials by considering oxygen concentrations. The author discusses the increase in hardness and Young’s modulus with oxygen concentrations under various conditions in detail. 

The following two papers in the reference list discuss nonlinear acoustic probing.

Acoustic probing is a well-established technique. Acoustic waves pass through most materials in a wide range of frequencies. This feature is contrastive to electromagnetic waves. One drawback is its wavelength. The typical acoustic frequency used for probing is several GHz, whereas the acoustic velocity in most materials is less than ten km/s. The corresponding acoustic wavelength is of the order of microns. This situation limits the size of anomalies to detect. A solution to this problem is the use of the nonlinear response of the material to the probing signal. The following two papers discuss nonlinear acoustic probing.

Cho et al. [5] used the second harmonic of an acoustic emitter signal in their probing system operating in the pulse-echo mode. A challenge in such nonlinear techniques is the smallness of the signal received by the sensor. To overcome this issue, the authors of this paper considered intensifying the acoustic signal by focusing the acoustic beam from the emitter. In this paper, they propose an annular phased-array acoustic transducer and demonstrate the increase in the nonlinear sensitivity via numerical analyses.

High acoustic coupling between the acoustic transducer and the specimen is a significant factor for successful probing. Distilled water and acoustic gel are common materials for coupling. Usually, these materials increase the coupling efficiency so that the acoustic signal detected by the receiver is reasonably high. However, the receiver signal depends on the contact pressure between the transducer and the specimen, compromising the repeatability of data. 

In ref. [6], Jeong et al. propose a method to evaluate the nonlinear parameter with air-coupled acoustic receivers. By developing a two-layer acoustic model consisting of the solid specimen and air, considering diffraction and attenuation, they constructed an algorithm to evaluate the nonlinear parameter relative to a reference specimen. In this paper, they verified the algorithm via comparisons with the experiment. This paper also discusses factors that affect the accuracy of the method. 

Multiple methods applied to characterize the same specimen always provide extra information. In the following two papers, the authors take advantage of it. 

Niccolini et al. [7] investigated the critical behavior of fracture processes in rocks and cement-based materials by acquiring acoustic emission and electrical resistance time series. They conducted compression experiments using rod specimens of Luserna stone and cement mortar. The experimental results indicate that the change in electric resistance precedes the acoustic emission in all tested specimens. The authors ascribe this observation to the fact that the acoustic emission signal is related to larger cracks than the electric resistance signal. In this paper, they discuss other findings in terms of the fracturing process.

Residual stresses are difficult to analyze because they hide in the material. In many situations, it is necessary to alter their status for the observer to evaluate them. On the other hand, too much alteration would change the stress, compromising the measurement. This nature makes nondestructive estimation of residual stress challenging. Murata et al. [8] demonstrated a nondestructive method of residual stress analysis. They applied laser spot heating to residually stressed specimens and probed the relaxation to estimate residual stress. Using a thermal camera, they monitored the thermal process. In addition, by mechanically applying initial loads to the specimen, they made quantitative analyses. The authors verified the results via comparisons with strain gauge measurements.

The recent advancement in software, generally called machine learning algorithms, facilitates the existing technology of deformation analyses. Regardless of the choice of hardware, the output signal representing deformation is complicated. Often, the information is buried in noise, and its spatiotemporal characteristics are complex. This situation causes human operators to make errors in diagnosis. Computers are capable of analyzing a large volume of data and extracting features. 

In the following three papers, the authors utilize the advantages of computer-based algorithms to deal with complex data.

Takahashi et al. [9] used an optical interferometric method called Electronic Speckle-Pattern Interferometry (ESPI) to discuss the dynamics of plastic deformation and fracture under tensile loading. The ESPI technique reveals the contour of differential displacements (the displacement occurring during a short time interval) as dark fringes in a full-field optical image.

For the analysis of deformation dynamics, it is necessary to analyze numerous fringe images. An algorithm to automatically locate the fringes is essential. Using speckles, the optical image containing the differential displacement contour is noisy. Takahashi et al. applied Gaussian filtering to reduce the noise and located the fringes continuously until the specimen fractured. With this method, they found that in the transition from late plastic deformation to the fracturing stage, the rotational mode of deformation plays a significant role in the process of stress concentration.

Kopfler et al. [10] use optical speckles for another purpose. They utilized the randomness of the speckle pattern to uniquely identify local areas of a transparent specimen (polyethylene films) and analyzed nonuniform deformation under tensile loading. With the numerical algorithm known as Digital Image Correlation, they found the nonuniform displacement field of the specimen. In the cases where the deformation is more uniform, they analyzed the deformation in the frequency domain using Fast Fourier Transform.

Barile et al. [11] propose an acoustic emission-based analysis of the damage progression stage in Sintered Laser Melting (SLM) materials. Characterization of materials processed by SLM is of great interest but also challenging due to the intrinsic orthotropy of these materials. When tested by acoustic emission methods, the orthotropic structure of the material causes the spectral content of the resulting waveforms to depend on the nature of the source and the building direction of the materials. In this view, the authors have taken advantage of machine learning tools and specifically of a convolutional neural network to classify detected signals based on the damage stage and the building direction. In particular, by adopting k-fold cross-validation, they have demonstrated drastic improvement in classification accuracy. 

Finally, Pavlovskii et al. [12] reviewed the recent progress and challenges in organic anode materials for Lithium-Ion Batteries (LIB), one of the most demanding technologies of the modern era. This review compares the electrochemical performances of different organic anode materials, discussing the advantages and disadvantages of each class of organic materials in research and commercial applications. After addressing the practical applications of some organic anode materials, the paper discusses some techniques to address significant issues, including low discharge voltages and the undesired dissolution of the anode material into electrolytes.
